# Myocardial work - correlation patterns and reference values from the population-based STAAB cohort study

**DOI:** 10.1371/journal.pone.0239684

**Published:** 2020-10-08

**Authors:** Caroline Morbach, Floran Sahiti, Theresa Tiffe, Vladimir Cejka, Felizitas A. Eichner, Götz Gelbrich, Peter U. Heuschmann, Stefan Störk, On behalf of the STAAB consortium

**Affiliations:** 1 Comprehensive Heart Failure Center, University Hospital and University of Würzburg, Würzburg, Germany; 2 Department of Medicine I, Cardiology, University Hospital Würzburg, Würzburg, Germany; 3 Institute of Clinical Epidemiology and Biometry, University of Würzburg, Würzburg, Germany; 4 Clinical Trial Center, University Hospital Würzburg, Würzburg, Germany; Universita degli Studi di Roma La Sapienza, ITALY

## Abstract

**Background:**

Recently, *myocardial work* analysis as an echocardiographic tool to non-invasively determine LV work has been introduced and validated against invasive measurements. Based on systolic blood pressure and speckle-tracking derived longitudinal strain (GLS) during systole and isovolumic relaxation, it is considered less load-dependent than LV ejection fraction (LVEF) or GLS and to integrate information on LV active systolic and diastolic work.

**Objectives:**

We aimed to establish reference values for global constructive (GCW) and global wasted work (GWW) as well as of global work index (GWI) and global work efficiency (GWE) across a wide age range and to assess the association with standard echocardiography parameters to estimate the potential additional information provided by myocardial work (MyW).

**Methods:**

The *Characteristics and Course of Heart Failure STAges A/B and Determinants of Progression* (STAAB) cohort study carefully characterized a representative sample of the population of the City of Würzburg, Germany, aged 30–79 years. We performed *myocardial work* analysis using the standardized, quality-controlled transthoracic echocardiograms of all individuals lacking any cardiovascular risk factor.

**Results:**

Out of 4965 participants, 779 (49±10 years, 59% women) were eligible for the present analysis. Levels of GCW, GWW, and GWE were independent of sex and body mass index, and were stable until the age of 45 years. Thereafter, we observed an upward shift to further stable values of GCW and a linear increase of GWW with advancing age, resulting in lower GWE. Age-adjusted percentiles for GCW, GWW, GWI, and GWE were derived. Higher levels of blood pressure or LV mass were associated with higher GCW, GWI, and GWW, resulting in lower GWE; higher LVEF correlated with higher GCW and GWI, but lower GWW. Higher E/e´ correlated with higher GWW, higher e´ with lower GWW.

**Conclusions:**

Derived from a large sample of apparently healthy individuals from a population based-cohort, we provide age-adjusted reference values for myocardial work indices, applicable for either sex. Weak correlations with common echocardiographic parameters suggest MyW indices to potentially provide additional information, which has to be evaluated in diseased patient cohorts.

## Introduction

In-depth knowledge of the biomechanic properties of the left ventricle (LV) is a prerequisite for understanding the pathophysiology of left ventricular (LV) dysfunction. Invasive assessment of the intracardiac LV pressure-volume relationship is the gold standard to determine LV stroke work, a measure that comprehensively describes how “hard” the LV is working [1]. Only recently, an echocardiographic method to non-invasively determine LV stroke work, called *myocardial work*, has been introduced and validated against invasive measurements [2–4]. The method is based on speckle-tracking derived longitudinal strain and peripheral systolic blood pressure. As such, *myocardial work* is thought to be less load-dependent compared to LV ejection fraction or longitudinal systolic strain [5]. Further, the new method allows us to differentiate myocardial constructive from wasted work components, thus offering new insights into cardiac mechanics and the pathophysiology of cardiac disease states.

Estimation of pathologic conditions requires the definition of normal. Since the method has been introduced only recently, there is still insufficient information on the performance of *myocardial work* characteristics in healthy individuals. We therefore aimed 1) to establish reference values for *global constructive* and *global wasted myocardial work* of healthy individuals derived from a large, well characterized, population-based cohort and 2) to further characterize this new echocardiographic tool by assessing the association of constructive and wasted work with age, sex, anthropometry, and echocardiographic parameters.

## Methods

### Study population and recruitment

We present an analysis of the *Characteristics and Course of Heart Failure Stages A-B and Determinants of Progression* (STAAB) Cohort Study, based on consecutive participants from the general population of Würzburg, Germany, aged 30–79 years and stratified for age and sex. The detailed study design and methodology has been published [6]. All study related procedures are subjected to a rigid and regular quality control process [6]. The STAAB cohort study protocol and procedures received positive votes from the Ethics Committee of the Medical Faculty (vote 98/13) as well as from the data protection officer of the University of Würzburg (J-117.605-09/13). All participants provided written informed consent prior to any study examination.

### Cardiovascular risk factors

History of arterial hypertension, dyslipidemia, diabetes mellitus, cardiovascular disease (previous myocardial infarction, coronary artery disease, stroke, peripheral artery disease), and current pharmacotherapy was assessed by physician-led face-to-face interview. Assessment of smoking status, height, weight, and blood pressure (sitting position after five minutes rest), and an oral glucose tolerance test were performed according to standard operating procedures by trained and certified personnel [6]. Fasting lipid profile and glycosylated hemoglobin (HbA1c) were measured at the central laboratory of the University Hospital Würzburg. Cardiovascular risk factors were defined according to current recommendations as follows: hypertension [7], blood pressure ≥140/90 mmHg or anti-hypertensive pharmacotherapy; dyslipidemia, low density lipoprotein ≥190 mg/dl [8] or lipid-lowering pharmacotherapy; obesity [9], body mass index >30 kg/m^2^; diabetes mellitus [10], HbA1c >6.5% or fasting plasma glucose >7.0 mmol/l or 2h-plasma glucose >11.1 mmol/l or anti-diabetic medication; smoking, current or ex-smoker.

### Echocardiography

All patients underwent an extensive, pre-specified transthoracic echocardiography protocol performed by dedicated trained personnel that was internally certified and quality-controlled in 6-month intervals [11]. A Vivid S6 scanner with a M4S sector array transducer (1.5–4.3 MHz, GE Healthcare, Horten, Norway) or a Vivid E95 scanner with a M5SC-D transducer (1.5–4.6MHz; GE Healthcare, Horten, Norway) was used. A minimum of three cardiac cycles was recorded. Two-dimensional images from the LV apical four-, two-, and three-chamber views were recorded with a frame rate of 50 to 80 s^-1^ and stored digitally. We measured LV end-diastolic and end-systolic volumes and calculated LV ejection fraction (LVEF; Simpson´s biplane method). Valve regurgitation was determined integrating the color Doppler multiplane vena contracta method and the pressure half time method, and valve stenosis was quantified assessing maximal flow velocity by continuous-wave Doppler according to current recommendations [12, 13].

### Myocardial work analysis

All *myocardial work* analyses have been performed by one single person (FS). To assess intra-observer variability, 20 random scans were read by one person (FS) twice, >2 weeks apart, for inter-observer variability, the same scans were read by a second person (CM) blinded to the previous results.

**Fig 1:**
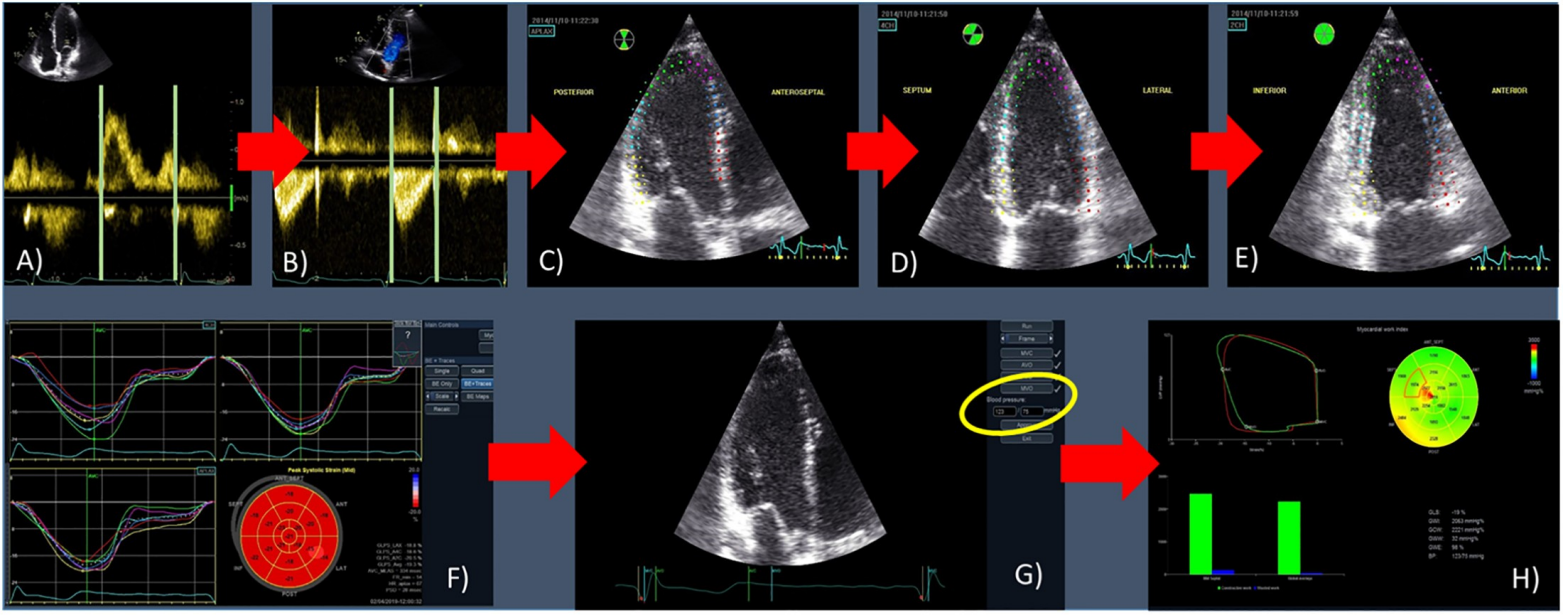
Determination of *myocardial work*. For exact timing, mitral valve opening and closure are determined using a pulsed-wave Doppler recording of the mitral inflow (A). Aortic valve opening and closure are determined using a continuous-wave Doppler recording through the aortic valve (B). Time points might be adjusted in the apical three chamber view (D), if necessary. Using *automated function imaging*, global longitudinal strain is determined in the apical three (C), four (D), and two (E) chamber views. The automated speckle tracking contour can be adjusted manually. After completion of strain determination (F), systolic and diastolic blood pressure levels have to entered (G) in order to determine global and segmental *myocardial work* (H).

Fig 1 exemplifies the determination of myocardial work. For timing of aortic and mitral valve closure and opening, we used continuous-wave Doppler through the aortic valve and pulsed wave Doppler of the mitral valve inflow. As changes in heart rate during the examination might affect the loop area, we visually verified these time points in the apical three-chamber view and manually adjusted them, when necessary. LV apical four-, two-, and three-chamber views were analyzed off-line using integrated software (Automated Functional Imaging; EchoPAC^®^, Version 202, GE) to determine global longitudinal peak systolic strain (GLS). After entering brachial systolic blood pressure values the software calculates constructive and wasted work. Constructive work describes the net effect resulting from positive work (shortening) performed during systole plus negative work (lengthening) performed during isovolumic relaxation. Wasted work describes the net effect resulting from negative work (lengthening) performed during systole plus positive work (shortening) during isovolumic relaxation. By aggregating the segmental values for constructive and wasted work (18-segment model), the software calculates global constructive (GCW) and global wasted work (GWW) as mean of the respective segmental values. For the current analysis, LV segments with poor tracking or suboptimal image quality were excluded as were subjects whose echocardiograms provided information on GLS and *myocardial work* in less than 17 segments. The software further provides a global work index (GWI = total work performed = area of the pressure-strain loop) and the global work efficiency (GWE = GCW/(GCW+GWW)).

### The subgroup of the present analysis

For the determination of reference values, we defined a sub-sample of apparently healthy individuals, i.e., subjects free from cardiovascular risk factors and cardiovascular disease. We further excluded individuals with LVEF<50%, regional wall motion abnormalities, other than sinus rhythm or significant LV valve disease (any stenosis or > mild regurgitation of the mitral or aortic valve).

### Data analysis

Statistical analysis was performed using SPSS (Version 25 and 26, SPSS Inc., Chicago, USA). Descriptives of quantitative data are provided as mean and standard deviation. Regarding the definition of cardiovascular risk factors, missing values were treated as pathologic findings (i.e., individuals with missing information did not enter the apparently healthy subgroup), except for individuals with one missing blood glucose value (fasting glucose, 2h glucose, or HbA1c) in case of two valid and normal values. P-values <0.05 were considered statistically significant. Observer variability was assessed using Bland-Altman 95% limits of agreement. The distributions of GCW and GWW were analyzed in an explorative manner. Scatter diagrams of GCW or GWW with age were plotted with trend curves obtained from locally weighted regression, and general linear models were computed. Standard deviations of residuals in subgroups were compared by Levene’s test. Normality of residuals was examined by the Shapiro-Wilk test. Eventually, age-dependent percentiles were computed from the most suitable model for the estimated mean GCW or GWW, adding the respective percentile of the residuals. The association of GCW and GWW with anthropometry and echocardiographic measures was assessed using Kendall´s τ correlation coefficient.

## Results

**Fig 2:**
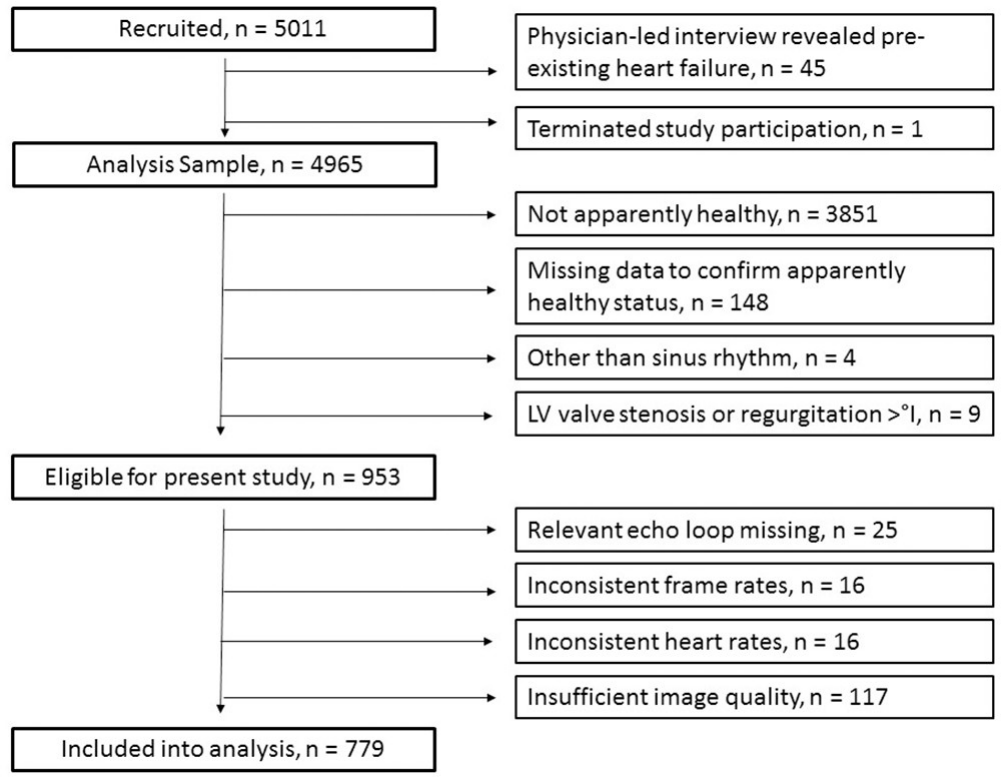
Study flow.

The STAAB cohort study recruited an age- and sex-stratified population-based sample of 5011 participants. In 45 participants, the physician-led interview revealed a pre-existing heart failure, hence, these individuals had to be excluded from further study participation and did not enter any analysis. Further, one participant terminated the study participation and did not enter any analysis, too. Of the remaining 4965 participants, n = 779 were eligible for the present analysis (Table 1 & Fig 2). The inter- and intraobserver variability of myocardial work parameters was favourably low (Table 2).

**Table 1:** Clinical characteristics of apparently healthy individuals with valid myocardial work analysis, and subgroups women vs men.

	Total	Men	Women	P
	N = 779	N = 322	N = 457	
Age [years], mean (SD)	49 (10)	49 (11)	49 (10)	0.87
Age groups [years], n(%)				
30-<39	128 (16)	55 (17)	73 (16)	
40-<45	146 (19)	61 (19)	85 (19)	
45-<50	167 (21)	70 (22)	97 (21)	
50-<55	118 (15)	45 (14)	73 (16)	
55-<60	83 (11)	32 (10)	51 (11)	
60 -<65	70 (9)	27 (8)	43 (9)	
65-<79	67 (9)	32 (10)	35 (8)	
BMI [kg/m^2^], mean (SD)	23.7 (2.7)	24.4 (2.4)	23.3. (2.8)	<0.001
BSA [m^2^], mean (SD)	1.82 (0.19)	1.98 (0.15)	1.71 (0.13)	<0.001
Systolic BP [mmHg], mean (SD)	119 (11)	124 (9)	115 (11)	<0.001
Diastolic BP [mmHg], mean (SD)	73 (8)	74 (8)	72 (8)	<0.001
Heart rate [min^-1^], mean (SD)	63 (9)	60 (9)	64 (9)	<0.001
Total cholesterol [mg/dl], mean (SD)	200 (34)	197 (31)	202 (36)	0.09
LDL [mg/dl], mean (SD)	115 (32)	120 (29)	112 (34)	0.001
Fasting glucose [mmol/l], mean (SD)	5.29 (0.53)	5.35 (0.54)	5.25 (0.52)	0.01
2h glucose [mmol/l], mean (SD)	5.62 (1.24)	5.43 (1.26)	5.77 (1.20)	0.001
HbA1c [%], mean (SD)	5.32 (0.34)	5.30 (0.34)	5.33 (0.35)	0.33
**Echocardiography**				
LVEDVi [ml/m^2^], mean (SD)	54 (11)	59 (12)	51 (10)	<0.001
LVMi [g/m^2^], mean (SD)	68 (14)	76 (14)	62 (12)	<0.001
LAVi [ml/m^2^], mean (SD)	22 (6)	23 (6)	21 (5)	0.003
LVEF [%], mean (SD)	61.1 (4.0)	60.8 (3.8)	61.4 (4.1)	0.06
E/e´, mean (SD)	6.7 (1.6)	6.5 (1.5)	6.9 (1.7)	0.001
E´, mean (SD)	11.1 (2.4)	10.8 (2.2)	11.3 (2.5)	0.002

Values are given as mean ± standard deviation (SD). P values refer to the comparison of men vs. women. Valid information for the respective parameters was >95% except for 2h glucose (n = 564, men n = 241, women n = 323), LVMi (n = 734, men n = 302, women n = 432), and LAVi (n = 643, men N = 265, women n = 378).

BMI = body mass index, BSA = body surface area, BP = blood pressure, HDL = high density lipoprotein, LDL = low density lipoprotein, HbA1c = hemoglobin A1c, LVEDVi = left ventricular enddiastolic volume index, LVMi = left ventricular mass index, LAVi = left atrial volume index, LVEF = left ventricular ejection fraction, E = early mitral inflow velocity, e´ = PW-Doppler derived early diastolic myocardial lengthening velocity (mean of septal and lateral wall).

**Table 2:** Observer variability for parameters describing myocardial work.

		Intra-observer variability		Inter-observer variability	
	Mean (SD)	Mean difference (SD)	95%CI of differences	Mean difference (SD)	95%CI of differences
**GCW** [mmHg%]	2532 (472.0)	26.4 (47.4)	4.2; 48.5	231.4 (199.8)	137.9; 324.9
**GWW** [mmHg%]	87.0 (44.2)	7.7 (18.5)	-1.0; 16.3	9.9 (49.4)	-33.0; 13.2
**LVEF** [%]	61.1 (4.8)	0.6 (3.2)	-0.9; 2.0	2.5 (5.2)	-0.0; 5.0
**GLS** [%]	-21.4 (2.3)	0.3 (0.3)	0.1; 0.5	1.9 (1.8)	1.1; 2.7

To assess intra-observer variability, 20 random scans were read by one person twice (FS), >2 weeks apart, for inter-observer variability, the same scans were read by a second person (CM) blinded to the previous results.

GCW = global constructive work: work performed during shortening in systole and adding negative work during lengthening in isovolumic relaxation.

GWW = global wasted work: negative work performed during lengthening in systole adding work performed during shortening in isovolumic relaxation.

SD = standard deviation, CI = confidence interval, LVEF = left ventricular ejection fraction, GLS = global longitudinal systolic strain.

### Impact of age and sex on GCW and GWW

**Fig 3:**
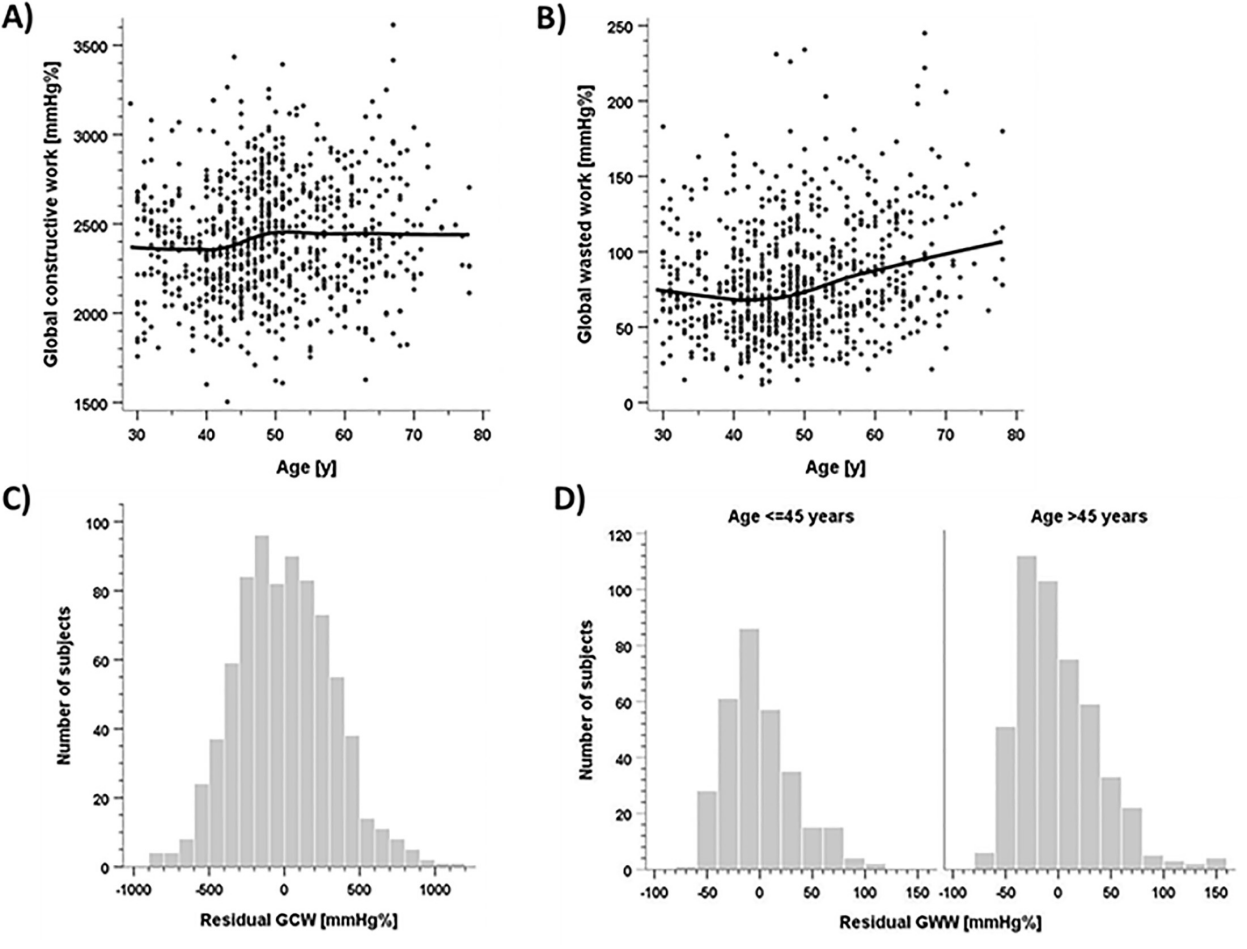
Association of global constructive (GCW, A) and global wasted (GWW, B) myocardial work with age and distribution of GCW (C) and GWW values (D). The curves were obtained from locally weighted regression analysis.

The plots of GCW and GWW by age are displayed in Fig 3A and 3B. Trend curves suggested altered dependencies around the age of 45 years. For further analysis we thus accepted the assumption of a modified association of age with myocardial work below vs above the threshold of 45 years. The respective numeric results are displayed in Table 3.

**Table 3:** Reference values for global constructive (GCW) and global wasted (GWW) myocardial work as well as for myocardial work index (GWI) and myocardial work efficiency (GWE).

		Age ≤45 years	Age >45 years			
		N = 304	N = 475			
**A) Global constructive work (GCW) [mmHg%]**						
Model with two slopes						
Mean at 45 years		2418				
Change per +10 years		+51	+25			
Test for slope		P = 0.11	P = 0.13			
Model with threshold at 45 years						
Mean		2366	2457			
95% CI		2330 to 2402	2428 to 2486			
Comparison of means		P<0.001				
SD of residuals		310	327			
Comparison of SDs		P = 0.11				
Common estimate of SD		320				
Test for sex difference		P = 0.16				
Normality of pooled residuals		P = 0.02				
2 outliers removed		P = 0.09				
** Percentiles**	**2.5**	**1739**	**1830**			
	**10**	**1956**	**2047**			
	**25**	**2150**	**2241**			
	**50**	**2366**	**2457**			
	**75**	**2582**	**2673**			
	**90**	**2776**	**2867**			
	**97.5**	**2993**	**3084**			
**B) Global wasted work (GWW) [mmHg%]**						
Model with two slopes						
Mean at 45 years		71				
Change per +10 years		−5	+14			
Test for slope		P = 0.15	P<0.001			
Model with one slope						
Estimated mean, ≤45 years		73				
Increase per 10 years, >45 years			+12			
95% CI of estimates		70 to 76	+9 to +16			
SD of residuals		33	38			
Comparison of SDs		P = 0.007				
Test for sex difference		P = 0.72				
Normality of residuals		P<0.001	P<0.001			
			**50y**	**55y**	**60y**	**65y**
** Percentiles**	**2.5**	**23**	**24**	**30**	**36**	**42**
	**10**	**35**	**36**	**42**	**48**	**54**
	**25**	**50**	**52**	**58**	**64**	**70**
	**50**	**68**	**73**	**79**	**85**	**91**
	**75**	**90**	**103**	**109**	**115**	**121**
	**90**	**119**	**131**	**137**	**143**	**149**
	**97.5**	**152**	**171**	**177**	**183**	**189**
**C) Global work index (GWI) [mmHg%]**		**Age ≤45 years**	**Age >45 years**			
Model with two slopes						
Mean at 45 years (men/women)						
Men		2174				
Women		2241				
Change per +10 years		+32	+2			
Test for slope		P = 0.27	P = 0.88			
(adjusted for sex)						
Model with threshold at 45 years						
Means						
Men		2141	2187			
95% CI		2099 to 2183	2149 to 2224			
Women		2206	2252			
95% CI		2168 to 2245	2220 to 2284			
Comparison >45 vs. ≤45 years		+46 (+2 to +89) P = 0.04				
(adjusted for sex)						
Comparison women vs. men		+66 (+22 to +108) P = 0.003				
(adjusted for age group)						
Test for sex by age interaction		P = 0.64				
SD of residuals						
Men		287	293			
Women		307	311			
Comparison of 4 SDs		P = 0.34				
Common estimate of SD		301				
Normality of pooled residuals		P = 0.03				
2 outliers removed		P = 0.09				
Percentiles						
Men	2.5	1551	1597			
	10	1755	1801			
	25	1938	1984			
	50	2141	2187			
	75	2344	2390			
	90	2527	2573			
	97.5	2731	2777			
Women	2.5	1616	1662			
	10	1820	1866			
	25	2003	2049			
	50	2206	2252			
	75	2409	2455			
	90	2592	2638			
	97.5	2796	2842			
**D) Global work efficiency (GWE) [%]**		**Age ≤45 years**	**Age >45 years**			
Model with two slopes						
Mean at 45 years		96.5				
Change per +10 years		+0.2	−0.6			
Test for slope		P = 0.14	P<0.001			
Model with one slope						
Estimated mean, ≤45 years		96.4				
Change per +10 years, >45 years			−0.5			
95% CI of estimates		96.3 to 96.5	−0.7 to −0.4			
SD of residuals		1.4	1.3			
Comparison of SDs		P = 0.01				
Test for sex difference		P = 0.42				
Normality of residuals		n.a.				
		**Age ≤45 years**	**>45–60 years**	**>60 years**		
Percentiles (raw)	2.5	93	92–93	91–92		
	10	94	93–94	92–93		
	25	95–96	94–95	93–94		
	50	96–97	95–96	95–96		
	75	96–97	96–97	96		
	90	97–98	97–98	96–97		
	97.5	98	97–98	97–98		

**A)** The trend curves of both GCW (cf. Fig 3A) and GWW (cf. Fig 3B) suggested changes of dependencies around the age of 45 years. The change in slopes at 45 years derived from a piecewise linear model for GCW was not significant. However, in a respective threshold model with the change of estimated mean GCW at 45 years, the difference of means was significant. Equal standard deviations in both age groups were thus accepted. Inclusion of sex into the model induced no significant changes of point estimates. Normal distribution of residuals was accepted after removing two outliers. Therefore, percentiles applicable for both sexes were computed assuming normal distribution and homogeneous standard deviations, with an upward shift occurring at the age of 45 years.

**B)** The change in slopes at 45 years derived from the piecewise linear model for GWW was significant. Therefore, the final model assumed a constant mean GWW until the age of 45 years, with a linear increase thereafter. The standard deviation of residuals was significantly higher in the age above 45 years. Again, no significant difference between sexes was found. As the assumption of normality of residuals was violated in both age groups, percentiles applicable for both sexes were based on non-parametric percentiles of residual distributions. Hence, these percentiles were different for age groups below and above 45 years with a continuous increase above the age of 45 years.

**C)** Like for GCW, the threshold model was adequate for GWI. In addition, women had significantly higher GWI values than men. As there was no significant interaction of sex and age group, the mean difference between sexes was assumed to be independent of age. The residuals were found to have homogeneous variances in the four groups defined by age and sex and to be normally distributed up to two outliers. Therefore, percentiles were derived from normal distributions.

**D)** Mean GWE was found to be constant up to the age of 45 years and then to decrease continuously. No significant difference between sexes was found. The variance was significantly higher in the younger subjects. Since the device provided only integer values of GWE, ranging from 91 to 99, the test for normality was not applicable, and percentiles were based on the raw data and not computed from a model. In order to reflect the decrease beyond 45 years, percentiles for two age groups in that range were presented.

When proceeding analysis with a piecewise linear model for GCW (accepting a change in slopes occurring at 45 years), both slopes did not achieve significance. A respective threshold model, however, yielded significant differences of means. Equal standard deviations in both age groups were therefore accepted (Table 3). Including sex into the model had no effect. Residuals were approximately normally distributed, and normality was formally accepted after removing two outliers. Therefore, percentiles applicable for either sex were computed assuming a normal distribution and homogeneous standard deviation, with an upward shift occurring at the age of 45 years. The reference values for GCW are presented in Table 3A. The median (quartiles) for individuals younger than 45 years was 2366 mmHg% (2150; 2582), and was 2447 mmHg% (2241; 2673) for individuals older than 45 years, respectively.

The piecewise linear model for GWW yielded a significant slope beyond the age above 45 years, but not below 45 years. Therefore, the final model assumed the median GWW remaining constant up to the age of 45 years, and increasing linearly thereafter. The standard deviation of residuals was significantly higher in individuals older than 45 years. Again, no significant influence of sex on these associations was found. Normality of residuals was violated in both age groups. Therefore, percentiles applicable for both sexes were based on non-parametric percentiles of residual distributions. The reference values for GWW are presented in Table 3B. The median (quartiles) for individuals younger than 45 years was 68 (50; 90) mmHg%, and was 73 (52; 103) mmHg% for individuals aged 50 years, respectively. With each subsequent decade, the median GWW increased by 6 points (Table 3B, bottom).

GWI showed higher values (+46 mmHg%) in individuals >45 years, when compared to younger participants with no further increase with advancing age. Women had significantly higher values (+66 mmHg%), when compared to men. Therefore, we provide sex-specific percentiles for individuals ≤ and >45 years (Table 3C).

GWE was not different between men and women and was stable until the age of 45 years. Thereafter, we found decreasing values with increasing age (-0.5% per decade, p for slope <0.001), hence we provide age-specific percentiles applicable for either sex (Table 3D).

### External factors affecting myocardial work indices

In a second step, we evaluated the association of GCW, GWW, GWI, and GWE with anthropometric and echocardiographic indices (Table 4). Because we had selected healthy individuals, these markers were all within normal ranges. Higher values of both systolic and diastolic blood pressure, but not of body mass index, were positively related with GCW, GWI, and GWW and negatively related with GWE. Higher LV ejection fraction and GLS were strongly associated with higher GCW, GWI, and GWE and with lower GWW. Further, higher LV mass was associated with both higher GCW and GWW, but lower GWE. Higher E/e´ was positively related to GCW and GWW, resulting in lower GWE, and e´ was inversely related to GWW, consecutively, higher e´ correlating with higher GWE. Larger LA volumes were associated with higher GCW. Stroke volume and heart rate showed no significant correlation with myocardial work indices (Table 4).

**Table 4:** Association (Kendall’s τ) of global constructive work (GCW), global wasted work (GWW), global work index (GWI), and global work efficiency (GWE) with anthropometrics and echocardiographic parameters of systolic and diastolic left ventricular function.

Variable	N	GCW		GWW		GWI		GWE	
		τ	P	τ	P	τ	P	τ	P
**Measurements associated with criteria for “apparently healthy”**									
Body mass index	779	0.00	0.99	−0.05	0.05	0.00	0.85	0.05	0.08
Systolic blood pressure	779	**0.38**	<0.001	**0.16**	<0.001	**0.35**	<0.001	**−0.09**	0.001
Diastolic blood pressure	779	**0.22**	<0.001	**0.12**	<0.001	**0.21**	<0.001	**−0.08**	0.001
Left ventricular ejection fraction	761	**0.13**	<0.001	**−0.12**	<0.001	**0.16**	<0.001	**0.18**	<0.001
Global longitudinal strain	779	0.44	<0.001	**−0.09**	<0.001	0.46	<0.001	0.22	<0.001
Left ventricular volume index	762	−0.01	0.57	0.04	0.13	−0.02	0.48	−0.04	0.16
Left ventricular mass index	739	**0.05**	0.04	**0.05**	0.03	0.02	0.50	**−0.06**	0.02
Left atrial volume index	643	**0.06**	0.02	−0.04	0.10	**0.06**	0.03	0.06	0.05
E/e’ (average)	760	**0.05**	0.05	**0.07**	0.003	**0.08**	0.002	**−0.08**	0.003
e’ (average)	761	0.01	0.68	**−0.15**	<0.001	**0.05**	0.04	**0.21**	<0.001
e’ (lateral)	757	0.00	0.98	**−0.14**	<0.001	0.04	0.09	**0.20**	<0.001
e’ (septal)	740	0.03	0.23	**−0.13**	<0.001	**0.07**	0.01	**0.19**	<0.001
Tricuspid regurgitation pressure gradient	288	**0.15**	<0.001	0.04	0.36	**0.17**	<0.001	−0.02	0.73
Stroke volume	761	0.02	0.51	−0.02	0.51	0.01	0.61	0.03	0.29
Heart rate	779	−0.01	0.72	0.03	0.15	−0.02	0.46	−0.03	0.20

E = pulsed-wave Doppler derived peak mitral inflow velocity, e´ = tissue Doppler and pulsed-wave Doppler derived mitral annular early diastolic relaxation velocity.

## Discussion

In a large sample of healthy individuals derived from a population-based cohort balanced for age and sex, we found myocardial work analysis an echocardiographic tool with good feasibility and a favorable intra- and inter-observer variability. GCW, GWW, and GWE were independent of sex and showed stable values up to the age of 45 years. Beyond that threshold, GCW and GWW behaved differently. Median GCW values showed a modest increment of about 4% around the age of 45 years, without major subsequent alterations at higher age groups. By contrast, GWW increased linearly with advancing age beyond the age of 45 years, resulting in decreasing GWE with advancing age. We here provided age-adjusted percentiles for both measures of myocardial work, which now may be used as reference for either sex. In contrast, GWI was higher in women when compared to men. In line with GCW, we found an increment in GWI around the age of 45 years with no further changes associated with advancing age.

As a consequence of the process of selecting participants for the current investigation, all anthropometric and echocardiographic measures were within normal ranges. Yet, we found disparate associations of anthropometry and LV geometry as well as of systolic and diastolic function with myocardial work indices. Higher LVEF and GLS were associated with higher GCW, GWI and GWE, and with lower GWW. Regarding diastolic function, we found higher E/e´ associated with higher GCW (trend), GWI, and GWW, but resulting in lower GWE, indicating that the increase in GWW with increasing filling pressure exceeds the increase in GCW. Further, higher e´ associated with lower GWW, which resulted in higher GWE, and larger LA volume was associated with higher GCW and GWI. Higher LV mass, as well as higher blood pressure, were associated with higher GCW but also higher GWW, consecutively resulting in lower GWE. Body mass index was not associated with myocardial work. In conclusion, systolic LV function correlated with GCW and GWI, while diastolic function correlated with GWW, consecutively both, systolic and diastolic function correlating with GWE.

To our knowledge, this is the first report providing reference ranges for non-invasively determined myocardial work parameters from healthy individuals over a wide age range derived from a large, well-characterized population-based cohort balanced for age and sex. The detailed cardiovascular characterization allowed identifying individuals without cardiovascular risk factors or known cardiovascular disease. Quality controlled, standardized echocardiography permitted to exclude individuals with valvular disease. Given that valid analysis of myocardial work requires three apical views in good image quality, the feasibility in our cohort was good. The semi-automated analysis showed good inter- and intra-observer variability, rendering myocardial work a reliable diagnostic tool.

While LVEF and GLS as measures of LV systolic function are known to show slightly more favorable values in women [14] myocardial work parameters, which, in addition to GLS, take systolic blood pressure into account, revealed no association with sex. This implies, that the real stroke work the myocardium has to perform, might be the same for either sex. Echocardiographic reference values are derived from healthy individuals and healthy women, who–as in our cohort–usually present with lower blood pressure values when compared to men [5]. Therefore, although the female myocardium appears to perform the same work but against a lower afterload, it might contract a little more, resulting in higher values of LVEF and more negative values of GLS. Thus, myocardial work might be the most reliable tool to study myocardial function independent of afterload conditions in either sex.

Interestingly, we found myocardial work stable until the age of 45 years. The moderate shift in GCW and GWI at higher age might be a result of changes in hormonal status with consecutive changes in blood pressure. Age-related changes in vascular function generally include deteriorating endothelial dysfunction and arterial stiffness, which is accompanied by increasing systolic blood pressure and pulse pressure even in individuals without cardiovascular risk factors. Below the age of 60 years, men compared to women exhibit a greater degree of endothelial dysfunction and worse arterial stiffness; beyond the age of 60, these vascular differences diminish. Below the age of 45 years, women have lower blood pressure than men, but blood pressure increases in the perimenopausal period. Subsequently, beyond the age of 64 years, the prevalence of hypertension is higher in women compared to men [15]. Regarding GWW, we found linearly higher values with higher age, potentially reflecting physiologic processes of healthy ageing like progressive fibrosis and modulation of cardiomyocytes. Consecutively, GWE decreased with advancing age.

Our results confirm but also extend previously published findings [5, 16]. Analyses from the EACVI Normal Reference Ranges for Echocardiography (NORRE) study [5] showed GCW higher in women when compared to men and higher in individuals >40 years when compared to younger adults. Regarding GWW, they found similar values for men and women and across all age groups [5]. Analyses from healthy study participants at the University Hospital of Rennes, France, showed higher GCW in women older than 35 years, but not in younger women when compared to men of same age. They further found no difference in GCW and GWW across the different age groups [16]. These incongruent findings might be due to sample size and the distribution of age and sex in the different cohorts (Tables 5 and 6). NORRE has the major strength of ethnic diversity of their study population which enables them to provide reference values valid for a large number of countries. On the other hand, a population based cohort study like STAAB with strict stratification for age and sex might due to its methodological approach be more appropriate to answer questions regarding sex- and age-dependency of echocardiographic measurements. Nevertheless, our results should be validated in different population-based cohorts from different countries and the evaluation of their physiological cause remains subject to further research.

**Table 5:** Comparison of population under study: STAAB vs NORRE [5] vs University Hospital of Rennes [1,6].

Characteristics	STAAB	NORRE	Rennes
**N, sites**	1	22	1
**Type of selection**	Population-based, stratified for age and sex	Convenience sample	Healthy cohort of different ongoing studies
**Inclusion criteria**	Age 30–79 years Citizen of Würzburg	Age ≥ 25 years Normal ECG	Age ≥ 18 years Normal ECG and physical examination
**Exclusion criteria**	Presence of CV risk factors (s) History of CV disease and/or heart failure Medical therapy with cardio-active drug Significant LV valve disease (mitral valve and aortic valve)	Presence of CV risk factor(s) History of CV disease Chronic exposure to excessive alcohol consumption Medical therapy with cardio-active drug Structural heart disease on echocardiogram	Presence of CV risk factors History of symptomatic cardiovascular or lung disease
**Echo machine**	GE Vivid S6 or E95	GE E9 or Philips IE33	GE, Vivid 7, Vivid E9, or E95
**N, total sample**	5000	734	N/A
**N, healthy participants**	779	734	N/A
**N, female participants**	457	320	N/A
**N, male participants**	322	414	N/A
**Age, years**	49 (10)	46 (13)	N/A
**BMI, kg/m**^**2**^	24 (3)	24 (3)	N/A
**BSA, m**^**2**^	1.8 (0.2)	1.8 (0.2)	N/A
**Systolic blood pressure, mmHg**	119 (11)	120 (13)	N/A
**Diastolic blood pressure, mmHg**	73 (8)	74 (9)	N/A
**Glucose level, mmol/l**	5.29 (0.53)	5.13 (0.67)	N/A
**Total cholesterol, mg/dl**	200 (34)	184 (31)	N/A
**LVEDVi, ml/m**^**2**^	54 (11)	51 (11)	N/A
**LVEF biplane, %**	61 (4)	64 (5)	N/A
**LAVi, ml/m**^**2**^	22 (6)	26 (6)	N/A

CV, cardiovascular; BMI- body mass index, BSA- body surface area, LVEDVi- left ventricular end diastolic volume index, LVEF- left ventricular ejection fraction biplane, LAVi- left atrial volume index (biplane).

**Table 6:** Comparison of individuals with valid myocardial work analysis: STAAB vs NORRE [5] vs University Hospital of Rennes [1,6].

	STAAB	NORRE	Rennes
**Number of healthy participants with MyW analysis**	779	226	115
**Number of female participants**	457	141	43
**Number of male participants**	322	85	72
**Age, years**	49 (10)	45 (13)	36 (13)
**Individuals age group 20–40**	128	95	<35 years n = 57
**Male/ Female**	55/73	N/A	N/A
**Individuals age group 40–60**	514	97	≥35 years n = 58
**Male/ Female**	208/306	N/A	N/A
**Individuals age group ≥ 60**	137	34	N/A
**Male/ Female**	59/78	N/A	
**BMI, kg/m**^**2**^	24 (3)	23 (3)	23 (3)
**BSA, m**^**2**^	1.8 (0.2)	1.8 (0.2)	1.8 (0.2)
**Systolic blood pressure, mmHg**	119 (11)	116 (12)	121 (8)
**Diastolic blood pressure, mmHg**	73 (8)	73 (8)	73 (6)
**Glucose level, mmol/l**	5.29 (0.53)	5.05 (0.61)	N/A
**Total cholesterol, mg/dl**	200 (34)	182 (31)	N/A
**GCW, mmHg%**	2430 (351)	2232 (331)	2224 (229)
**GWW, mmHg%**	74 (54–101)	78.5 (52–122)	90 (61–123)
**GWI, mmHg%**	2209 (307)	1896 (308)	1926 (247)
**GWE, %**	96 (95–97)	96 (94–97)	96 (94–97)

BMI- body mass index, BSA- body surface area, GCW = global constructive work, GWW = global wasted work, GWI = global work index (total work performed = area of the pressure-strain loop), GWE = global work efficiency (GCW/(GCW+GWW)).

Myocardial work indices were not associated with BMI and, taking afterload into account, are thought to be less load-dependent when compared to LVEF and GLS [5]. In addition to their sex-independency and their predictable age-dependency, this might make them a reliable and broadly applicable tool to assess myocardial function.

As expected, we found a positive association of GCW, GWI, and GWE and a negative association of GWW with LVEF. However, myocardial work indices also were associated with parameters of diastolic function. Integrating LV work during active relaxation in early diastole, myocardial work indices are the first, non-invasively obtained measures of almost total active LV work and might thus prove useful in the evaluation of the myocardial response to adverse cardio-metabolic conditions and cardiotoxicity. In addition, associations were only weak implying that MyW is likely to provide additional information beyond common echocardiographic parameters. The clinical and prognostic yield has to be evaluated in respective patient collectives.

### Strengths and limitations

We present data from a cross-sectional single-center study with predominantly Caucasian participants. Hence, reference values might have to be adjusted in individuals of different descent. For calculcation of MyW, we utilized blood pressure readings derived from brachial measurements, which may be considered reliable in non-diseased subjects. In patients, precision of MyW measurements may be improved employing central blood pressure. Further, the prognostic implication of potentially abnormal values can currently not be determined and remains subject to further research. Nevertheless, our results are derived from a population-based cohort exactly meeting the strict stratification criteria for age and sex, and detailed assessment of cardiovascular risk factors allowed to identify an apparently healthy sub-cohort of substantial size. The ongoing STAAB follow-up with serial echocardiography and standardized assessment of cardiovascular events is likely to give detailed insights into the course of cardiac function and the prognostic implications of constructive and wasted myocardial work.

## Conclusion

In healthy individuals from the general population, echocardiographically derived GCW, GWW, and GWE were independent from sex and BMI, but revealed a characteristic and disparate association with advancing age. In combination with its low load-dependency, high feasibility and low observer variability, non-invasively assessed *myocardial work* holds promise as a reliable non-invasive diagnostic tool. *Myocardial work* integrates LV work performed in systole and isovolumic relaxation. It is thus the first measure of almost total active myocardial function and might aid the assessment of adverse cardio-metabolic states or cardiotoxicity. Further, the differentiation of myocardial active work in constructive and wasted work offers the opportunity to evaluate the impact of cardiovascular risk factors and diseases on different aspects of myocardial performance.
